# The role of psychosocial stressors in PTSD development among medical staff in Ukraine

**DOI:** 10.1192/j.eurpsy.2025.277

**Published:** 2025-08-26

**Authors:** K. Golonka, S. Tukaiev, J. M. A. Ferreira, B. Palamar, K. Sitnik-Warchulska, D. Fortuna

**Affiliations:** 1 Institute of Applied Psychology, Jagiellonian University, Kraków, Poland; 2 Institute of High Technologies, Taras Shevchenko National University of Kyiv, Kyiv, Ukraine; 3 Faculdade de Medicina, Universidade de Coimbra, Coimbra, Portugal; 4 Department of Social Medicine and Public Health, Bogomolets National Medical University, Kyiv, Ukraine

## Abstract

**Introduction:**

Prevalence of posttraumatic stress disorder (PTSD) has been documented in war-affected populations. A third of Ukrainians met diagnostic requirements for PTSD according to the UN, but the information on the mental health of medical staff is still insufficient. Well-known increased risk of mental disorder is the result of traumatic experiences in healthcare workers as part of their professional duties.

**Objectives:**

Based on this we aimed to analyze the potential sources of traumatic experience at work and the severity of PTSD and depression symptoms among medical staff in war condition.

**Methods:**

The online and paper-pencil survey was conducted in November 2023 - January 2024. The study included a sample of 96 health care workers (doctors, nurses and paramedics). We used the structured interview on work characteristics of physical and psychosocial factors and standardized questionnaires on PTSD (Posttraumatic Stress Disorder Checklist; PCL-5) and depression (Patient Health Questionnaire; PHQ-9) symptoms. Additionally the needed source of support to cope with stress at work has been investigated.

**Results:**

The correlation and regression analysis allowed revealing patterns between potential sources of traumatic experience at work and the severity of PTSD and depression symptoms among Ukrainian medical staff. The results of the correlation analysis of physical and psychosocial stressors, depression and PTSD symptoms indicated that psychosocial stressors define PTSD development. There were detected job-related specific psychosocial stressors among medical staff in Ukraine leaded to PTSD and depression development: “Conflict in organization/community”, “Being threatened/abused”, “Lack of appreciation by the organization/community in which you work”.

**Conclusions:**

The analysis identified the most important factors (psychosocial stressors) determining levels of PTSD in medical personnel at war. These data contribute to a significant debate on the negative role of job conditions at war for health care workers.

**Disclosure of Interest:**

None Declared

